# Risk indicators in families with abused children and young people: *scoping review*

**DOI:** 10.1080/07853890.2021.1896244

**Published:** 2021-09-28

**Authors:** Aida de Jesus Correia Simões, Maria da Saudade Lopes

**Affiliations:** a Centro de Investigação Interdisciplinar Egas Moniz (CiiEM), Egas Moniz Cooperativa de Ensino Superior, Caparica, Portugal; b Escola Superior de Saúde do Politécnico de Leiria, Almada, Portugal

## Abstract

**Introduction:**

The promotion of rights and the protection of children implies involvement between state agencies and families in the evaluation processes, having developed in the child Welfare systems Internationally [1–3]. Although effective involvement is an essential component of the Help process [4], it presents continuous challenges for Professionals [5]. There is also an underlying tension between the regulatory role inherent in the protection system and the importance of involvement and contribution to develop the capacities of families producing better outcomes for children [6]. It is a challenge for families and professionals to deal with the duality of the relationship, given the expectations that workers engage in conflicting roles of supporting families, on the one hand, and ensuring the safety of children, on the other, having the authority and The mandate to remove children when necessary causes a level of mistrust that interposes in interactions [7]. We define as objectives to carry out a comprehensive research in order to identify how the families of children and young people in danger are evaluated and which indicators are used in the literature and in the organisations of the various countries. This *scoping review* presents as a premise the question of research: what are the family hazard indicators that jeopardise the harmonious development of children and young people? Scope studies present an increasingly frequent option to synthesise health evidences [8].

**Materials and methods:**

This scoping review was performed by both authors and a protocol was defined using the items for scoping review (PRISMA-SCR) Checklist, supported in two research strategies. A first through electronic databases EBSCOhost (CINAHL, Nursing & Allied Health Collection: Comprehensive, MEDLINE Complete, MedicLatina), BVS (MEDLINE, LILACS, Index psychology-technical-scientific journals, BDENF-nursing) and in a Second phase on the sites of State institutions and non-governmental organisations, which promote rights and protect children, with relevant research data (web of Science). The research was carried out between 2017 and 2018. All researches that could answer the research question were included, the limitation of this study was the difficulty in finding authors whose research is centre in the indicators of family hazard for children and young people, with a The vast literature where risk and danger coexist. The first author performed the research in the databases and on the websites of the organisations and the two authors examined the same (no 225) publications, discussed the results and defined the data to be harvested. The disagreements in the selection of studies and data extraction were resolved by consensus and discussion. The descriptors (keywords) and their combinations were: indicators, social service/social work/protection service, family/caregiver, negligence/abuse/maltreatment, child/Young, assessment/evalution tools. The data protocol to be extracted was jointly developed by the two reviewers who mapped the data independently, discussed the results and continuously updated the data collection protocol in an interactive process [9]. **Results**: Throughout the research process, we were confronted with the meaning of the concepts of risk and danger. For some realities it is understood as a risk the probability of an event happening, while danger is the condition for the risk to occur, however, most countries consider the danger as being a situation where the medium risk and high Risk. In this sense, the existence of a situation affecting the fundamental rights of the child is not sufficient; It is necessary for it to be unprotected in the face of this danger. What guided the identification of the indicators of family hazard, focussed on the absence of protective factors, which expose the child to the circumstances before which it is unprotected. After analysing the integral texts, seventeen articles fulfilled the criteria for inclusion in this study, immersed 35 family hazard indicators, as well as the danger problems for children associated with each of these, the findings were in a framework in order to mirror its representation. The sites included in this research also fulfilled these inclusion criteria, referring to hazard indicators, resulting from structured investigations and manuals, which serve as a reference for professionals, from a given Region or country. The results inherent to them were also structured in a framework that would render the collection objective and easily identifiable, We idenfied twenty-five indicators of family hazard. The risk indicators as a whole resulted, either from the research carried out in the databases or from the websites consulted. In the table below are listed all the indicators of family hazard that emerged from the literature (Table 1). **Discussion and Conclusions:** The studies included in a meta-analysis on the impact of family intervention programs at home in reducing neglect and ill-treatment for children considered that alcohol and/or drug abuse is an indicator of high risk in the perpetuation of Negligence in particular with children between 0 and 5 years, because it is considered a greater vulnerability in this age group [10]. One of the questions that is observed in the assessments performed are the attitudes of caregivers in relation to the child, and the negative attitudes towards the child are considered as a predictor of danger [11]. In order to identify the detailed risk profile of the families referred to the CPS, it was included as an indicator for a high risk of negligence, mothers living in poverty, having been enrolled to receive home visits until the child completes Five years [12]. Loman and Siegel found significant differences between parental competence impairment, when physical abuse is in question (the child suffered severe harm that was inflicted on him) and when the problem signalled is neglect Children (lack of supervision, unmet basic needs, unsafe domestic environment and absence of health care) [4]. There is no evidence that safety risks and domestic appearance are associated with the severity of child abuse potential, however, domestic safety and appearance factors are relevant in caregivers referenced as negligent for With their children and who have a substance abuse behaviour [13]. The father's criminal history was also identified as a significant risk factor regardless of the level of parental involvement in these high-risk families [12]. The recurrence of maltreatment (represented by the presence of multiple reports to the CPS) may be a more appropriate way to stratify the families involved with the child protection agencies. The recurrence of mistreatment was consistent with the number of times the family was signalled. Previous studies suggest that the number of reports and evidence of the reports should be considered as indicators of the severity of the abuse [12]. The home intervention programs reported by Cassillas et al. concentrate their attention mainly on mistreatment, having as target population parents with a history of maltreatment [10]. The verification of the full reports of the CPS, carried out by Duffy, Hughes, Asnes and Leventhal (2015), provided systematic information on the factors considered to increase the risk of child abuse the existence of a history of signalisation to Parent CPS as children. In the investigation carried out by the aforementioned authors, almost half of the mothers had a history of previous involvement with the CPS, considering this situation as one of the highest risk levels for the mothers [12]. The age of caregivers (adolescents/older age) is one of the reasons, either for their signalling to services (CPS) or for intervention programs with home follow-up conducted by Community agencies [10]. As a risk factor in relation to social characteristics, social deprivation is mirrored, as well as a social network Parca [12]. For Cassillas, Fauchier, Derkash and Garrido (2016) being a single parent was considered a risk factor for the existence of maltreatment and neglect [10]. The only risk factor that was of borderline significance for the outcome of recurrence was the presence of many caregivers. In this study, this finding was potentially important about the care environment in these high-risk families and need more studies where this variable is included [12]. There is a comprehensive literature on risk factors for abuse and neglect, in the four major risk domains, one of the identified are the social characteristics, in which the violent neighbourhood encompasses, as well as police involvement and Feeling of insecurity [12]. The caregiver's physical health is considered as a safety item, hence its absence increases the danger situation for the child. The history of psychiatric illness is one of the risk factors referenced, and maternal sociopathy is one of the mental problems encountered where the dangerousness increases [4]. Based on the several studies conducted over decades, they were focussed as one of the six risk factors for the absence of mental health of caregivers. It is verified in the reports of the families referred to the CPS, the highest levels of danger for the mothers, essentially centred on mental health problems [12]. Domestic violence is present in almost all families referred to the services, being considered one of the factors for the existence of danger for children. According to Loman and Siegel, there were no differences in the case of domestic violence (measured by acts of family violence, violence around the child and threats of care) The families referred to the services (CPS) have a high percentage of Episodes of violence. The authors found that violent behaviour around the child and acts of family violence jeoparthe their safety in the immediate [4]. Investment in children involves a whole range of services, but also regulates the behaviour and responsibilities of professionals and parents. This is a future-oriented approach, investing in children is designing the assurance that later develop productive and law-abiding adults. States will have to assume a new proactive and preventive role mainly because the current challenges are great, "the family" is not considered the most appropriate, by itself, to accomplish the expected tasks. This may have also been one of the historical motivations for the challenges in changing child protection systems [14]. We found that the indicators of family hazard are centred on several factors that contribute to the severity of the maltreatment inflicted on children and young people, it is unanimous for several authors the existence of alcohol and drug consumption; Mental illness; Domestic violence; Adverse socio-economic conditions; Parental competence. Most countries lack an adequate response, which respects the child's time, being the whole context of ambiguous evaluation and intervention, poorly structured and with scarce longitudinal studies that allow us to verify which strategy is most appropriate for Each of the situations referenced. We still have a long way to go in order to fulfil the entire convention of the rights of our children and young people.

**Table 1.** Family hazard indicators that emerged from *scoping review*.

1	Alcohol and/or drug abuse
2	Negative attitudes towards the child
3	Lack of social support
4	Absence of transport
5	Low schooling of caregivers
6	Low economic resources/long-term unemployment/no financial autonomy
7	Commitment of parental competencies/discipline rigid, inflexible or inconsistent/recourse to physical punishment
8	Inappropriate communication between separate parents/separations and conflictual divorces
9	Conditions/security/instability/housing precharity
10	Caregiver with criminal record/history of crime
11	Multiple journeys to emergency services with the child
12	Divergences/conflicts/dysfunctional/family isolation
13	Existence of Stepmothers and stepfathers
14	Unrealistic expectations of the caregiver for the child
15	Lack of co-operation of the caregiver with the services
16	Families already referenced to protection services
17	Single-parent families/unmarried mothers
18	Perpetuating history of inflicting ill-treatment
19	Personal history of having been the victim of maltreatment/abandonment in childhood
20	Parental signalling history as a child
21	Age of caregivers (adolescents/advanced age)
22	Multiple Caregivers
23	Child's special Needs
24	High number of children in family
25	Unwanted child
26	Physical health problems
27	Mental Health problems
28	Parents absent in children's education
29	Being the first child
30	Domestic violence
31	Violence in the community and in the neighbourhood/social neighbourhoods overcrowded/environmental negligence/neighbourhoods with crime/victims of discrimination
